# Perceived loneliness and social support in bipolar disorder: relation to suicidal ideation and attempts

**DOI:** 10.1186/s40345-024-00329-8

**Published:** 2024-03-20

**Authors:** Chelsea K. Pike, Katherine E. Burdick, Caitlin Millett, Jessica M. Lipschitz

**Affiliations:** 1 Department of Psychiatry, Brigham and Women’s Hospital, 221 Longwood Avenue, Boston, MA 02115 USA; 2 Department of Psychiatry, Harvard Medical School, Boston, MA USA; 3 Northwell, New Hyde Park, NY USA

**Keywords:** Bipolar disorder, Loneliness, Social support, Suicide

## Abstract

**Background:**

The suicide rate in bipolar disorder (BD) is among the highest across all psychiatric disorders. Identifying modifiable variables that relate to suicidal thoughts and behaviors (STBs) in BD may inform prevention strategies. Social connectedness is a modifiable variable found to relate to STBs in the general population, but differences exist across subgroups of the general population and findings specifically in BD have been equivocal. We aimed to clarify how perceived social connectedness relates to STBs in BD.

**Method:**

146 adults (86 BD, 60 healthy controls) completed clinical interviews (Hamilton Depression Rating Scale; Structured Clinical Interview for DSM-5) and self-report measures of loneliness (UCLA Loneliness Scale) and social support (Interpersonal Support Evaluation List). Analyses explored differences in indicators of social connectedness (loneliness and social support) between BD participants and healthy controls, and explored relationships between STBs (lifetime suicide attempts and current suicidal ideation) and indicators of social connectedness in BD participants.

**Results:**

BD participants reported significantly higher loneliness and lower social support than healthy controls. In BD participants, perceived social support was significantly related to both ever having attempted suicide and number of lifetime attempts. Interestingly, perceived loneliness, but not social support, was significantly associated with current suicidal ideation.

**Conclusions:**

Findings expand the evidence base supporting a relationship between perceived social connectedness and STBs in BD. They suggest that this modifiable variable could be a fruitful treatment target for preventing STBs in BD.

## Background

The rate of suicide in bipolar disorder (BD) is 20–30 times greater than in the general population (Plans et al. 2019) and is among the highest across all psychiatric conditions (Miller and Black 2020). Between 4% and 19% of BD patients die by suicide (Dome et al. 2019), up to 50% attempt suicide at least once in their lifetime, and approximately 79% experience suicidal ideation during the depression phase of the disorder (Miller and Black 2020). Therefore, it is crucial to identify factors related to suicidal thoughts and behaviors (STBs) in BD, particularly those that may be modifiable through treatment.

Social connectedness is a modifiable variable that has been found to be relevant to suicide risk. Specifically, a recent meta-analysis identified loneliness as a predictor of future STBs (McClelland et al. 2020). However, this meta-analysis also indicated that heterogeneity existed across studies and highlighted that social connectedness may be stronger or weaker for specific subgroups. Within the subgroup of patients with BD, the relationship between STBs and social connectedness has been relatively under-examined (Miller and Black 2020). Two studies have found a relationship between suicide attempts and living alone (Arici et al. 2018; Hansson et al. 2018). Studies have also explored the relationship between perceived social support and STBs, but findings have been mixed. One study found that lower perceived social support was related to higher levels of current suicidal ideation (Xie et al. 2018). However, other work found that perceived social support at baseline did not directly predict changes in later ideation (Owen et al. 2022), and that there was no difference in perceived social support between those with and without a prior suicide attempt (Studart et al. 2016). Thus, within the subgroup of bipolar disorder, further exploration of the relationship between social connectedness and STBs is warranted.

Clarifying the relationship between perceived social connectedness and STBs may be particularly important in the context of COVID-19. Research consistently indicates that patients with BD exhibit lower perceived social connection than healthy controls (Eidelman et al. 2012; Fowler and Dooley 2023; Furukawa et al. 1999; Xie et al. 2018). Many studies have reported an increase in loneliness since the start of the COVID-19 pandemic in accordance with social distancing regulations (Buecker and Horstmann 2022). Now, over three years into the pandemic, changes to social patterns that may impact social connectedness continue. Many individuals have shrunken social networks and/or are more selective in their social activities to minimize risk during high-COVID months, making feelings of isolation more common than they were pre-pandemic. As such, loneliness and social support may be especially important variables to target when seeking to reduce STBs in BD.

The current study extends previous research on social connectedness and STBs by examining the relationship between social connectedness and STBs in this high-risk subgroup (i.e., patients with BD). Goals were to evaluate (a) whether expected differences in self-reported social connectedness between those with BD and healthy controls were replicated in our sample and (b) the relationship between self-reported social connectedness and STBs in BD participants, controlling for depression severity. We hypothesized that social connectedness of those with BD and healthy controls would be significantly different, and that, in BD participants, low social connectedness would be associated with lifetime suicide attempt and current suicidal ideation even when controlling for depression severity.

## Methods

### Participants

Participants were part of a multi-year longitudinal study of BD. Recruitment for this study was via hospital Listservs, patient registries, and other studies of BD within the Mood and Psychosis Research Program at Brigham and Women’s Hospital. Inclusion criteria for BD participants were: (1) age 18–68, (2) bipolar I disorder (BD-I) or bipolar II disorder (BD-II) diagnosis per the Structured Clinical Interview for DSM-5 (SCID-5), and (3) affective stability at baseline per the Clinical Global Impressions Scale-Bipolar Version (Spearing et al. 1997). Exclusion criteria were: (1) history of central nervous system trauma, (2) diagnosed neurological disorder, (3) attention-deficit hyperactivity disorder treated in childhood prior to BD diagnosis, or known learning disability, (4) diagnosed mild cognitive impairment or dementia, (5) substance misuse or dependence within the past 3 months, (6) active, unstable medical problem that may interfere with cognition, and (7) electroconvulsive therapy in the past year. Matched healthy controls meeting all above criteria, except without an Axis I disorder (per SCID-5), were also recruited.

Data was collected from a total of 146 participants (86 BD and 60 demographically-matched healthy controls). This represented a subset of participants in the longitudinal study because loneliness and social support measures were only added to the protocol midway through the parent study. Of the participants who completed the social connectedness measures, one was excluded owing to not providing data on STBs. Additionally, seven participants were excluded because they did not complete the social connectedness measures even after they had been added to the protocol. The majority of participants’ baseline visit data was used, but for a small number of early participants (*n* = 18), data from 9-, 18-, or 27-month visits was used. The Massachusetts General Brigham Human Research Committee reviewed and approved all study procedures (Approval Number: 2019P000419). All participants provided informed consent prior to their participation.

### Measures

#### Sociodemographics

Sociodemographic items asked about gender, age, race, and years of education.

#### Suicide attempts and ideation

Number of lifetime suicide attempts was assessed using the SCID-5. The 24-item Hamilton Depression Rating Scale (HAM-D; Kovacs 1981) scale was administered to participants in order to assess depression severity in the past several days. Item 3 of the HAM-D specifically probes suicidal ideation on a scale of 0 to 4. A score of 0 was taken to indicate “no suicidal ideation,” and scores of 1 or higher were considered to indicate suicidal ideation, as done in prior studies (Park et al. 2014; Pu et al. 2015). In analyses utilizing this indicator of suicidal ideation, depression was accounted for by including the total HAM-D score minus the participant’s score on HAM-D Item 3.

#### Perceived social connectedness

*Revised UCLA Loneliness Scale (R-UCLA).* The R-UCLA (Russell et al. 1980), is a 20-item self-report questionnaire designed to measure trait-level subjective feelings of loneliness and social isolation. Participants rate each item on a four-point Likert scale from 1 (never) to 4 (often). Higher total scores indicate greater feelings of loneliness. Previous research indicates that the R-UCLA has good reliability and validity (Russell et al. 1980; Knight et al. 1988).

*Interpersonal Support Evaluation List (ISEL).* The ISEL (Cohen et al. 1985) is a 12-item self-report questionnaire designed to measure perceived social support. Participants rate each item on a four-point Likert scale from 1 (definitely false) to 4 (definitely true). Higher total scores indicate higher levels of perceived social support. The ISEL has been shown to have good construct validity and internal consistency (Cohen 2008; Merz et al. 2014).

### Statistical analyses

Analyses were performed with IBM SPSS Statistics, Version 28.0.0.0. Descriptive statistics for the BD and healthy control groups were calculated, expressed as means and standard deviations for continuous variables, and percentages for non-continuous variables. One-way analysis of variance was used to look for differences in R-UCLA and ISEL score between the BD and healthy control groups. All analyses utilized cross-sectional data.

Among those with BD, binary logistic regression was used to examine whether R-UCLA and ISEL scores differed between those with and without lifetime suicide attempts and current suicidal ideation, when controlling for depression severity (per HAM-D). Multiple linear regression was also performed to assess the relationship between number of lifetime suicide attempts and R-UCLA and ISEL scores, controlling for depression severity (per HAM-D).

## Results

Table 1 summarizes sociodemographic characteristics and descriptive data for the clinical and self-report measures used. Consistent with previous research, one-way analysis of variance indicated that, compared to healthy controls, BD participants had significantly higher R-UCLA scores (*F*(1,141) = 49.253, *p* < .001) and significantly lower ISEL scores (*F*(1,138) = 29.410, *p* < .001), indicating greater loneliness and lower levels of social support. These differences remained significant even after controlling for age and gender.

**Table 1:** Descriptive statistics

	Measure	N	Min.	Max.	Mean	SD	%
**Bipolar**	Age (years)	86	19	66	40.73	14.43	-
	Education (years)	86	11	24	16.15	2.41	-
	Gender (% female)	86	-	-	-	-	74.42
	Race (% white)	86	-	-	-	-	79.07
	BD-I (%)	86	-	-	-	-	75.58
	HAM-D score	86	0	37	7.65	7.58	-
	R-UCLA score	86	20	74	44.40	12.88	-
	ISEL score	81	13	48	34.47	8.59	-
**Healthy Controls**	Age (years)	60	22	68	45.92	14.61	-
	Education (years)	60	12	22	16.66	1.90	-
	Gender (% female)	60	-	-	-	-	58.33
	Race (% white)	60	-	-	-	-	76.67
	HAM-D score	58	0	3	0.28	0.77	-
	R-UCLA score	57	20	61	30.65	8.91	-
	ISEL score	59	25	48	41.49	5.86	-

Note. HAM-D = Hamilton Depression Rating Scale; R-UCLA = Revised UCLA Loneliness Scale; ISEL = Interpersonal Support Evaluation List; Min. = minimum value; Max. = maximum value; SD = Standard Deviation

Results of all regression analyses are presented in Table 2. Logistic regressions controlling for depression severity indicated that BD participants with a lifetime suicide attempt reported lower levels of social support than those without a lifetime attempt and those experiencing current suicidal ideation reported higher levels of loneliness than those without current ideation. Linear regressions indicated that those with a greater number of lifetime suicide attempts reported lower levels of social support, controlling for depression severity.

**Table 2:** Regressions evaluating associations between social connectedness and STBs in BD controlling for depression severity

**Logistic regressions:**	**Lifetime Suicide Attempt Status**						
	*n*	B	SE	OR	CI	Wald	*p*
R-UCLA	84	0.04	0.02	1.04	1.00–1.08	3.66	0.056
ISEL	79	− 0.07	0.03	0.93	0.88–0.99	5.28	0.022*
**Linear regressions**:	**Number of Lifetime Suicide Attempts**						
	*n*	B	SE	β	Corr.	*F*	*p*
R-UCLA	84	0.01	0.02	0.10	0.09	0.34	0.412
ISEL	79	− 0.05	0.02	− 0.25	0.24	2.37	0.032*
**Logistic regressions**:	**Current Suicidal Ideation Status**						
	*n*	B	SE	OR	CI	Wald	*p*
R-UCLA	86	0.08	0.04	1.08	1.01–1.16	4.57	0.033*
ISEL	81	− 0.07	0.05	0.93	0.85–1.02	2.40	0.121

Note. R-UCLA = Revised UCLA Loneliness Scale; ISEL = Interpersonal Support Evaluation List; CI = Confidence Interval; Corr. = zero-order correlations; *p ≤ .05

## Discussion

This study found a connection between perceived social connectedness and STBs in BD. Perceived social support was significantly related to both ever having attempted suicide and number of lifetime suicide attempts. Interestingly, perceived trait-level loneliness, but not perceived social support, was significantly associated with current suicidal ideation.

These results add to the existing literature in several ways. First, findings replicate the previously observed difference in perceived social connection between healthy controls and patients with BD. Second, our findings expand upon the literature supporting an association between perceived social connectedness and STBs. While this picture appears to be relatively clear in the general psychiatric population, findings within BD, where suicide rates are much higher, have been equivocal. Some studies report a relationship between social support and STBs (Xie et al. 2018) and others found no association (Owen et al. 2022; Studart et al. 2016). Third, findings suggest that there may be some nuance to the way social connectedness impacts STBs. Specifically, the experience of being a lonely person may be more strongly related to current thoughts of suicide, whereas the perception of having others that are supportive (whether or not one feels lonely) may be more strongly related to whether you act on these thoughts. Another possible explanation of our results is that past attempts are detrimental to supportive social relationships, which do not necessarily recover and this is reflected in the observed connection between past attempts and current social support. While the broad takeaway is that interventions aimed at improving social connectedness may have a positive impact on STBs in BD, this also points to the importance of identifying the direction of observed relationships. For example, if suicide attempts erode social support, interventions focusing on rebuilding social support after attempts may be particularly important.

There are several limitations to this study. First, sample size may have limited the ability to obtain significant findings for some of the relationships investigated. Replication in a larger sample would be useful. Second, use of a larger sample would have allowed us to further sub-divide into population subgroups within BD, such as gender or BD-I versus BD-II, where findings may have been weaker or stronger. This will be an important future step and may explain some of the conflicting findings in BD. Third, this paper uses cross-sectional data, and the cross-sectional associations observed do not indicate causality. Future research evaluating the longitudinal relationship between social connectedness and STBs will be required to make causal inferences. Fourth, all of the data for this study was collected during the COVID-19 pandemic, but after vaccines became available. While the measures of social connectedness used in this study were designed to measure trait-level perceived social connectedness, social distancing guidelines extending past the availability of vaccines could have impacted feelings of social connectedness. Finally, this was a secondary analysis of a sample that was not recruited for this purpose. Accordingly, the sample was restricted based on the eligibility criteria established by the parent study, which may limit generalizability, especially to BD patients who are in a less stable phase of the disease. Nonetheless, this sample provided a rich opportunity to evaluate the relationship between social connectedness and STBs.

## Conclusions

This study sheds light on the relationship between social connectedness—a potentially modifiable variable—and STBs in BD patients. Findings support the importance of social connectedness as a protective factor for STBs in patients with BD. Furthermore, since we evaluated social connectedness from different angles—both perceived loneliness and perceived social support—findings suggest that there may be nuance to the way social connectedness impacts STBs. Specifically, whereas high loneliness may be more heavily linked to thoughts of suicide, low social support may be more heavily linked to suicide attempt. Findings support further investigation into the nuances of the relationship between perceived social connectedness and STBs in BD and highlight the potential impact of interventions directly targeting factors like interpersonal effectiveness or social skills training in this sample.

## Data Availability

We are not currently able to share data because data collection is still ongoing (with findings presented based on interim analyses) and the study is not yet federally funded. The informed consent used in this study allows for data sharing and we do expect to share our data once data collection is complete and a data repository is in place.

## Abbreviations

BD: Bipolar disorder
STBs: Suicidal thoughts and behaviors
SCID-5: Structured Clinical Interview for DSM-5
HAM-D: Hamilton Depression Rating Scale
R-UCLA: Revised UCLA Loneliness Scale
ISEL: Interpersonal Support Evaluation List
